# Supplementary material for: A Novel, Nontoxic and Scalable Process to Produce Lipidic Vehicles
Source: Materials (Basel). 2020 Nov 8;13(21):5035. doi: 10.3390/ma13215035 (PMC7664659; doi:10.3390/ma13215035)
Supplement: Supplementary file 1 [file materials-13-05035-s001.zip › materials-980596-supplementary.docx]

Supplementary Materials: A Novel, Nontoxic and Scalable Process to Produce Lipidic Vehicles

Nikolaos Naziris, Natassa Pippa and Costas Demetzos *

Section of Pharmaceutical Technology, Department of Pharmacy, School of Health Sciences, National and Kapodistrian University of Athens, Panepistimioupolis Zografou, 15771 Athens, Greece; niknaz@pharm.uoa.gr (N.N.); natpippa@pharm.uoa.gr (N.P.)

***** Correspondence: demetzos@pharm.uoa.gr; Tel.: +30‑21‑0727‑4596

| 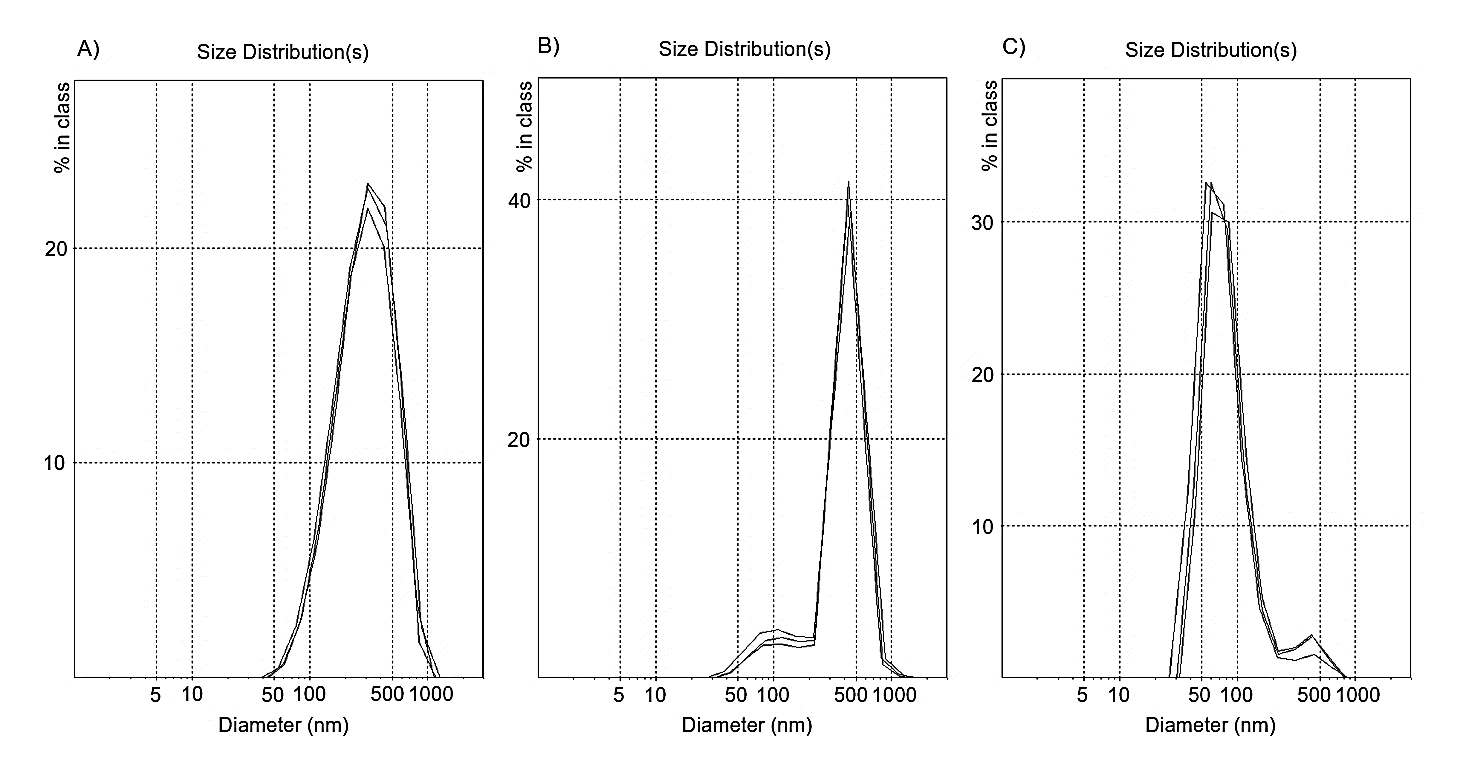 |
| --- |
| (**a**) |
| 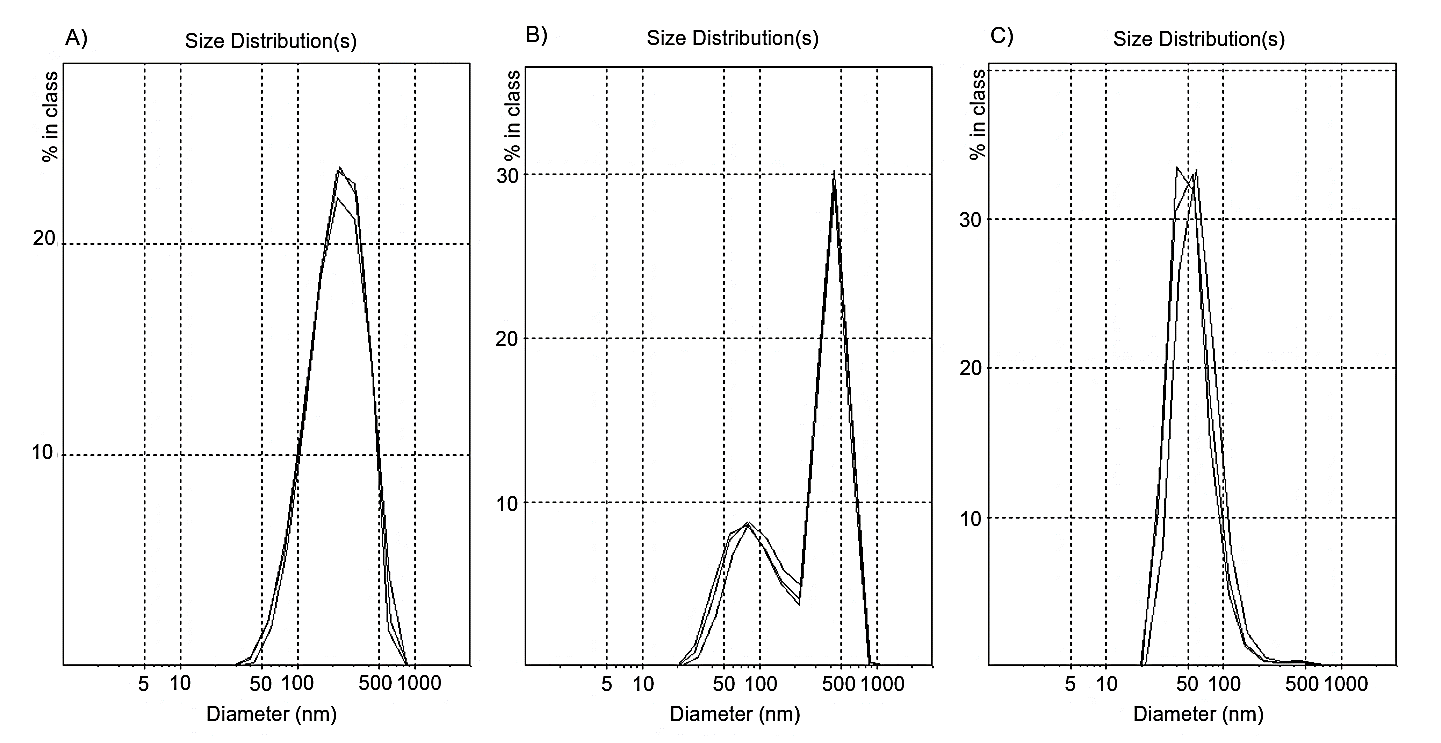 |
| (**b**) |

**Figure S1:** Distribution by (A) intensity (DI), (B) volume (DV) and (C) number (DN) of (**a**). HSPC:SA 9:0.25 and (**b**). EggPC:CHOL:SA 9:1.8:0.25 lipidic vehicles with glycerine concentration 20% *v/v*, built in Table 2. Colloidal stability diagram in terms of particle size (hydrodynamic diameter, *D_h_*) of systems with incorporated curcumin HSPC:SA:CUR 9:0.25:0.8 and 9:0.25:1 with glycerine concentration 20% and 10% *v/v*, respectively.

**Figure S2:** Colloidal stability diagram in terms of particle size (hydrodynamic diameter, *D_h_*) of systems with incorporated curcumin HSPC:SA:CUR 9:0.25:0.8 and 9:0.25:1 with glycerine concentration 20% and 10% *v/v*, respectively.

**Table S1:** Peak analysis by intensity (*D_I_*), volume (*D_V_*) and number (*D_N_*) of particles for two systems with glycerine concentration 20% *v/v*.

| **System** | **Molar Ratio** | **Glycerine Concentration (%** *v/v***)** | **Hours at 90 °C** | **Analysis** | **Peak** | **Area (%)** | **Mean** | **Width** |
| --- | --- | --- | --- | --- | --- | --- | --- | --- |
| HSPC:SA | 9:0.25 | 20% | 2 | Intensity | 1 | 100.0 | 336.3 | 520.3 |
|  |  |  |  | Volume | 1 | 16.7 | 123.3 | 102.3 |
|  |  |  |  |  | 2 | 83.3 | 458.0 | 347.3 |
|  |  |  |  | Number | 1 | 96.5 | 77.3 | 64.1 |
|  |  |  |  |  | 2 | 3.5 | 441.1 | 178.5 |
| EPC:CHOL:SA | 9:1.8:0.25 | 20% | 2 | Intensity | 1 | 100.0 | 237.5 | 349.3 |
|  |  |  |  | Volume | 1 | 37.7 | 97.4 | 164.3 |
|  |  |  |  |  | 2 | 62.3 | 421.2 | 309.0 |
|  |  |  |  | Number | 1 | 99.4 | 55.1 | 45.3 |

**Table S2:** The physicochemical properties of lipidic vehicles before, after 1, 2, 5 and 10 passes of extrusion through polycarbonate filters.

| **System** | **Molar Ratio** | **Glycerine Concentration (% v/v)** | **Extrusion** | **D_h_ ^1^ (nm)** | **SD ^2^** | **PDI ^3^** | **SD** |
| --- | --- | --- | --- | --- | --- | --- | --- |
| HSPC:SA | 9:0.25 | 20% | Before | 245.6 | 3.5 | 0.302 | 0.013 |
| HSPC:SA | 9:0.25 | 20% | After 1 Pass | 141.6 | 3.2 | 0.232 | 0.009 |
| HSPC:SA | 9:0.25 | 20% | After 2 Passes | 179.3 | 3.7 | 0.209 | 0.013 |
| HSPC:SA | 9:0.25 | 20% | After 5 Passes | 147.8 | 2.9 | 0.170 | 0.027 |
| HSPC:SA | 9:0.25 | 20% | After 10 Passes | 146.4 | 2.9 | 0.179 | 0.017 |
| EPC:SA | 9:0.25 | 10% | Before | 353.5 | 5.5 | 0.627 | 0.032 |
| EPC:SA | 9:0.25 | 10% | After 1 Pass | 136.9 | 3.2 | 0.287 | 0.012 |
| EPC:SA | 9:0.25 | 10% | After 2 Passes | 106.1 | 2.1 | 0.287 | 0.005 |
| EPC:SA | 9:0.25 | 10% | After 5 Passes | 94.3 | 2.2 | 0.282 | 0.012 |
| EPC:SA | 9:0.25 | 10% | After 10 Passes | 84.6 | 2.0 | 0.307 | 0.002 |
| DPPC:SA | 9:0.25 | 15% | Before | 250.2 | 11.0 | 0.402 | 0.026 |
| DPPC:SA | 9:0.25 | 15% | After 1 Pass | 155.5 | 4.2 | 0.254 | 0.032 |
| DPPC:SA | 9:0.25 | 15% | After 2 Passes | 125.9 | 2.4 | 0.182 | 0.018 |
| DPPC:SA | 9:0.25 | 15% | After 5 Passes | 127.1 | 2.3 | 0.174 | 0.004 |
| DPPC:SA | 9:0.25 | 15% | After 10 Passes | 134.6 | 2.1 | 0.204 | 0.010 |

^1^ Hydrodynamic diameter; ^2^ Standard deviation; ^3^ Polydispersity index.

**Publisher’s Note:** MDPI stays neutral with regard to jurisdictional claims in published maps and institutional affiliations.

| 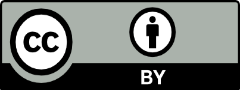 | © 2020 by the authors. Submitted for possible open access publication under the terms and conditions of the Creative Commons Attribution (CC BY) license (http://creativecommons.org/licenses/by/4.0/). |
| --- | --- |
